# *In vitro* and *in vivo* Activity of Phibilin Against *Candida albicans*

**DOI:** 10.3389/fmicb.2022.862834

**Published:** 2022-05-11

**Authors:** Zhongjie Li, Xiaoyuan Jing, Yaping Yuan, Yingbin Shui, Shasha Li, Zhuoran Zhao, Bo Deng, Wenlu Zhang

**Affiliations:** School of Basic Medical Sciences, Henan University of Science and Technology, Luoyang, China

**Keywords:** traditional Chinese medicine, antimicrobial peptide, *Candida albicans*, fungicidal, skin infection

## Abstract

The increase in the occurrence of antifungal-resistant *Candida albicans* infections necessitates more research to explore alternative effective and safe agents against this fungus. In this work, Phibilin, a new antimicrobial peptide obtained from *Philomycus bilineatus* and used in traditional Chinese medicine, effectively inhibits the growth and activities of *C. albicans*, including the clinical resistant strains. Phibilin is a fungicidal antimicrobial peptide that exhibited its antimicrobial effect against *C. albicans* mainly by disrupting the membrane and interacting with the DNA of the fungi. In particular, Phibilin induces the necrosis of *C. albicans via* the ROS-related pathway. Moreover, this antifungal compound inhibited the biofilm formation of *C. albicans* by preventing the development of hyphae in a dose-dependent manner. Furthermore, Phibilin and clotrimazole displayed a synergistic effect in inhibiting the growth of the fungi. In the mouse cutaneous infection model, Phibilin significantly inhibited the formation of skin abscesses and decreased the counts of *C. albicans* cells in the infected area. Overall, Phibilin is potentially an effective agent against skin infections caused by *C. albicans*.

## Introduction

*Candida albicans* is a ubiquitous commensal organism of the human microbiome that normally colonizes the skin, oral cavity, esophagus, gastrointestinal tract, and urogenital system. The fungus can cause several diseases, ranging from topical epithelial to severe systemic infections (Goffena et al., 2018; Niu et al., 2020). Also, infections caused by *C. albicans* are usually associated with high mortality (Wisplinghoff et al., 2004). Moreover, the burgeoning population of immunocompromised individuals and the growing use of invasive medical devices and implants have dramatically increased the incidence rates of *C. albicans* infections (especially nosocomial infections) (Ding et al., 2016). Although azoles, polyenes, and echinocandins are effective therapeutics against *C. albicans* infections, they can cause severe side effects (Shapiro et al., 2011). Furthermore, the overuse of these antifungal drugs has rapidly increased drug resistance and the incidence of resistant *C. albicans* infections (Brown et al., 2014). Therefore, it is imperative to explore novel and more effective agents against *C. albicans*.

Antimicrobial peptides (AMPs) are typically small, cationic, and amphipathic peptides (Zhang and Gallo, 2016). To date, more than 8,000 AMPs have been identified (antimicrobial peptide database CAMP)^1^. They exhibit a broad spectrum of antimicrobial activity against bacteria, fungi, and viruses, including drug-resistant strains (Li et al., 2020a). Given their broad antimicrobial action, AMPs have huge potential for therapeutic applications.

*Philomycus bilineatus* (also termed as *Limax* in traditional Chinese medicine) is a gastropod mollusk that is used in traditional Chinese medicine to treat various diseases, such as cancer, bronchial asthma, carbuncle, and erysipelas (Dictionary of Traditional Chinese Medicine) (Chen et al., 2004; Yan et al., 2011; Yang T. et al., 2019). The whole transcriptome of *P. bilineatus* was well studied based on the next-generation sequencing technology (Li et al., 2020b). In the present study, a novel AMP named as Phibilin was identified using *in silico* comparison of the transcriptome sequencing data of *P. bilineatus* and known AMP sequences from Collection of AntiMicrobial Peptides (CAMP) and Antimicrobial Peptide Database (APD) (Thomas et al., 2010; Yoo et al., 2014; Wang et al., 2016). The *in vitro* and *in vivo* activity and action mechanism of Phibilin against *C. albicans* were investigated in the present study.

## Materials and Methods

### Animals

The female BALB/c mice (20–30 g) were maintained under standard conditions of humidity (50 ± 5%), temperature (25 ± 2°C), and dark–light cycles (12 h each), with free access to food and water. The mice were humanely euthanized (anesthetized by intraperitoneal pentobarbital injection and sacrificed by cervical dislocation) at the end of the experiments.

### Peptide and Fungal Strains

The peptide Phibilin (RGDILKRWAGHFSKLL) used in this study was synthesized by GL Biochem (Shanghai, China) with amidated C-terminal ends. The purity of the peptide was more than 95%. *C. albicans* AY93025, *C. albicans* ATCC 10231, and *C. albicans* CMCC 98001 were procured from the China Center of Type Culture Collection, while the clinical resistant strains of *C. albicans* (Table 1) were obtained from the affiliated hospitals of the Henan University of Science and Technology and stored in our laboratory.

**TABLE 1:** Clinical resistant strains of *C. albicans* used in study.

Parameters	*C. albicans* CR-1	*C. albicans* CR-2	*C. albicans* CR-3	*C. albicans* CR-4	*C. albicans* CR-5
Amphotericin B	+	+	+	+	+
Nystatin	+	−	+	+	+
Caspofungin	+	+	+	−	−
Clotrimazole	−	+	−	+	+
Fluconazole	+	+	−	+	+

*The susceptibility testing was performed using the filter paper diffusion method based on CLSI guidelines. + : Sensitive; −: Resistance.*

### Secondary Structure Analysis

The secondary structure of Phibilin was determined using circular dichroism (CD) spectroscopy and analyzed using the Heliquest program^2^ (Li et al., 2014). The CD assays were performed on a J-810 spectropolarimeter (Jasco, Tokyo, Japan) at room temperature and the UV range of 190–250 nm. The peptide was prepared at a final concentration of 0.1 mg/ml in water, 25% trifluoroethanol (TFE)/H_2_O, 50% TFE/H_2_O, or 75% TFE/H_2_O. Spectra were collected from three separate recordings.

### Antifungal Activity

The antifungal activity of the peptide was determined using the broth microdilution assay, as recommended by the Clinical and Laboratory Standards Institute with minor modifications (CLSI, 2019). Briefly, the peptides were serially diluted in 0.9% saline, whereas the cells of *C. albicans* were diluted in the yeast extract peptone dextrose (YPD) medium to obtain a final concentration of 10^3^–10^4^ cells/ml at the exponential growth phase. Then, 40 μl of the peptide dilutions at varying concentrations and 160 μl of the cell dilutions were added to the sterile 96-well plates. The plates were incubated for 24 h at 35°C with continuous shaking at 200 rpm/min. The growth of the cells was determined by measuring the absorbance at 630 nm. The minimum inhibitory concentration (MIC) was the lowest concentration of the peptide that inhibited the growth of the *C. albicans* cells. To determine the minimum fungicidal concentration (MFC), 10 μl of broth was taken from all the wells with no visible growth of fungal cells and plated on the YPD agar plates. MFC was defined as the lowest concentration of the peptide that causes a ≥ 99.9% reduction in the number of colony-forming units (CFU) in comparison to the starting inoculum count. The experiment was repeated at least three times.

### Time-Killing Kinetics

The *in vitro* killing curve for the peptide was determined as previously described with minor modification (Gu et al., 2021). Briefly, *C. albicans* AY93025 cells at the exponential phase were diluted to 10^5^–10^6^ CFU/ml in the YPD medium and treated with 1 × MIC, 2 × MIC, or 4 × MIC of Phibilin, and saline was used as the negative control. The aliquots were collected at defined intervals, serially diluted in saline, and plated on YPD agar plates. The plates were incubated at 35°C for 24 h, after which the CFUs were counted. Triplicate samples were collected at each defined interval, and the experiment was repeated at least three times.

### PI Absorption Assay

The integrity of the fungal cell membrane was analyzed using the PI absorption assay (Wang et al., 2018). Briefly, *C. albicans* AY93025 cells (approximately 10^5^–10^6^ CFU/ml) at the exponential phase were incubated with the 1 × MIC or 2 × MIC of Phibilin for 30 min at 35°C. Saline was used as the negative control. Samples were then harvested through centrifugation, rinsed in saline, and incubated with PI at a final concentration of 5 μg/ml for 30 min at 35°C in the dark. Then, the samples were rinsed again with saline and observed under a ZEISS fluorescence microscope (Axio Observer 3, Germany), and the percentage of the fluorescence-positive cells was calculated. The experiment was repeated at least three times.

### Scanning Electron Microscopy

*Candida albicans* AY93025 cells (approximately 10^6^–10^7^ CFU/ml) at the exponential phase were incubated with 1 × MIC, 2 × MIC, or 4 × MIC of Phibilin for 30 min at 35°C. Saline was used as the negative control. After centrifugation, the cells were rinsed with saline, fixed for 1 h with 2.5% glutaraldehyde in PBS, dehydrated with increasing concentrations of ethanol (30, 50, 70, 90, and 100%), and air-dried at room temperature. The dry samples were then gold-coated using a K550X sputter coater (Quorum Technologies, United Kingdom) and then observed with an SEM (JSM-6390LV, Japan).

### DNA-Binding Assay

Approximately, 300 ng of the pET-28A plasmid DNA or linear single-stranded DNA of the salmon sperm was mixed with varying concentrations of Phibilin for 10 min at room temperature, followed by electrophoresis in 1% agarose gel (Yuan et al., 2022). The migration of DNA was detected under UV illumination using a Bio-Rad Gel Documentation system. The experiment was repeated at least three times.

### ROS Measurement

The production and accumulation of ROS in *C. albicans* cells were analyzed using 2′, 7′- dichlorofluorescein diacetate (DCFHDA) (Jia et al., 2018). *C. albicans* AY93025 cells (approximately 10^6^–10^7^ CFU/ml) at the exponential phase were incubated with 1 × MIC, 2 × MIC, or 4 × MIC of Phibilin for 2 h at 28°C. Saline was used as the negative control. The cells were harvested through centrifugation, washed, and resuspended in a YPD medium. DCFH-DA was added to a final concentration of 10 μM. Following a 30 min incubation in the dark, the fluorescence intensity of *C. albicans* cells was determined using a BD Accuri C6 flow cytometer (Bectone Dickinson, United States). The experiment was performed in triplicate and repeated at least three times.

### Mitochondrial Membrane Potential Assays

The change in mitochondrial membrane potential of *C. albicans* cells was analyzed using 5,5′,6,6′-tetrachloro-1,1′,3,3′-tetraethyl-benzimidazolyl carbocyanine iodide (JC-1; Molecular Probes) (Yang Y. et al., 2019). *C. albicans* AY93025 cells (approximately 10^6^–10^7^ CFU/ml) at the exponential phase were treated with 1 × MIC, 2 × MIC, or 4 × MIC of Phibilin for 2 h at 28°C. Saline was used as the negative control. The cells were harvested, washed, and stained with 2.5 μg/ml of JC-1 for 20 min in the dark. After rinsing in PBS, the cells were analyzed by flow cytometry. The ratio of the fluorescence intensities of aggregates of JC-1 (FL2) to monomers (FL1) was calculated. The experiment was performed in triplicate and repeated at least three times.

### Annexin V and PI Staining

*Candida albicans* AY93025 cells (approximately 10^6^–10^7^ CFU/ml) at the exponential phase were treated with 1 × MIC, 2 × MIC, or 4 × MIC of Phibilin for 2 h at 28°C. Saline was used as the negative control. The *C. albicans* cells were harvested through centrifugation and washed using PBS. Annexin V/PI assays were performed according to the staining protocol included in the kit (SolarBio Life Science). Then the cells were analyzed using flow cytometry. The experiment was performed in triplicate and repeated at least three times.

### Effects of Phibilin on Biofilm Formation

Briefly, 100 μl of *C. albicans* AY93025 cells at the exponential phase in the YPD medium (10^5^–10^6^ CFU/ml) were added to the sterile 96-well plates and incubated for 4 or 24 h at 35°C to allow biofilm formation at different stages. Thereafter, each well was washed three times using PBS to remove the non-adherent cells. Then, 100 μl of YPD broth medium containing varying concentrations of Phibilin was added into the biofilm-coated wells. After incubation for another 24 h at 35°C, the biofilms were examined under a ZEISS fluorescence microscope. The cell viability of the biofilms was measured using the XTT assay (Scarsini et al., 2015). The experiment was repeated at least three times.

### Effect of Phibilin on Hyphae Formation

The effect of Phibilin on the morphological transformation of *C. albicans* was investigated using RPMI 1,640 medium supplemented with 10% fetal bovine serum, and this medium is found to induce hyphae formation (Yang et al., 2018). *Candida albicans* AY93025 cells at the exponential phase were diluted in RPMI 1640 medium to obtain a final concentration of 10^5^–10^6^ CFU/ml. Cell suspension (100 μl) was transferred to a 96-well plate and treated with 1 × MIC, 2 × MIC or 4 × MIC of Phibilin, and saline was used as the negative control. The 96-well plate was incubated in a shaking incubator maintained at a constant temperature. The cell suspension was then aspirated and rinsed with 200 μl of PBS for three times to remove the non-adherent cells. Then, the cells were observed and photographed using a ZEISS fluorescence microscope. The experiment was repeated at least three times.

### Drug Interaction Interpretation

The interactions between Phibilin and other commercial antifungal drugs were investigated using the checkerboard assay based on the fractional inhibitory concentration index (FICI) model (Cassone and Otvos, 2010). The activity of serial dilutions of different antifungal agents was analyzed in the presence of a constant Phibilin concentration (one-quarter of the peptide MIC). The combination MIC (cMIC) of any given combination was measured as described in the MIC assay. The fractional inhibitory concentration index (FICI) was calculated according to the following equation: FICI = cMIC_A_/MIC_A alone_ + cMIC_B_/MIC_B alone_. Synergy was defined as FICI ≤ 0.5. Indifference or absence of interaction was defined as 0.5 < FICI < 4. Antagonism was defined as FICI > 4. The experiment was repeated at least three times.

### Hemolytic Activity

The normal mice were humanely euthanized (anesthetized by intra-peritoneal pentobarbital injection and sacrificed by cervical dislocation), and then the eyeballs were removed to collect the blood samples. Freshly extracted red blood cells (RBCs) from the blood samples were washed thrice with saline and resuspended in saline to obtain a final concentration of 2% (v/v). The cell suspension containing RBCs (100 μl) and serially diluted peptide solution (100 μl) were added to the 96-well plate. Each concentration was conducted in triplicates. For positive and negative controls, 1% Triton X-100 and saline were used, respectively. After incubation for 1 h at 37°C with gentle shaking, the plate was centrifuged at 1,000 *g* for 10 min. Then, 100 μl of the supernatant from each well was transferred to a new 96-well plate, and the absorbance was measured at 490 nm. The hemolysis percentage was calculated according to the following equation: Hemolysis% = (H sample - H negative)/(H positive - H negative) × 100%, where H is the absorbance at 490 nm. The experiment was repeated at least three times.

### Cytotoxicity Assay

The HEK293T and L02 cell lines were cultured in Dulbecco’s Modified Eagle’s Medium supplemented with 100 μg/ml streptomycin, 100 U/ml penicillin, and 10% (v/v) fetal bovine serum, and maintained in a humidified chamber at 37°C and 5% CO_2_. Then, 6,000 cells in 100 μl of the medium were pre-seeded in sterilized wells of 96-well plates and incubated for 24 h. Thereafter, 100 μl of the medium supplemented with different concentrations of the peptide was added. Saline and 0.1% Triton X-100 were the negative and positive controls, respectively. After incubation for another 24 h, 20 μl of MTS solution (Promega, G3582) was added to each well and incubated for an additional 1–4 h. The viability percentage was calculated according to the equation: Viability% = (A sample - A positive)/(A negative - A positive) × 100%, where A is the absorbance at 490 nm.

### Cutaneous Infection Model in Mice

A mouse subcutaneous infection model was developed as previously described with little modification (Garcia et al., 2019), which was used to evaluate the activity of Phibilin against *C. albicans in vivo*. Briefly, the fur on the back of each mouse was shaved, and the exposed skin was sterilized using 10% povidone/iodine solution and 70% ethyl alcohol. *C. albicans* AY93025 cells at the exponential phase were harvested, washed, and resuspended in saline. Then, 50 μl of the suspension (10^7^–10^8^ CFU/ml) was subcutaneously injected into the back of the mice near the tail. One hour after infection, 50 μl of Phibilin (500 μg/ml), positive control agent (clotrimazole, 500 μg/ml), or negative control agent (saline) was directly injected subcutaneously into the infected area (intra-abscess injection), once daily for 3 days. The mice were humanely euthanized on the 4th day. Then, the skin abscesses (including all accumulated pus) were excised, homogenized in saline using a stomacher, serially diluted, and cultured on YPD agar for 18–24 h at 35°C. The number of CFU per gram of the tissue was then calculated.

### Statistical Analysis

The data were analyzed using the GraphPad Prism 6 software. Continuous normally distributed data were expressed as the mean ± standard error of the mean. The differences between groups were analyzed using one-way ANOVA.

## Results

### The Secondary Structure of Phibilin

**FIGURE 1:**
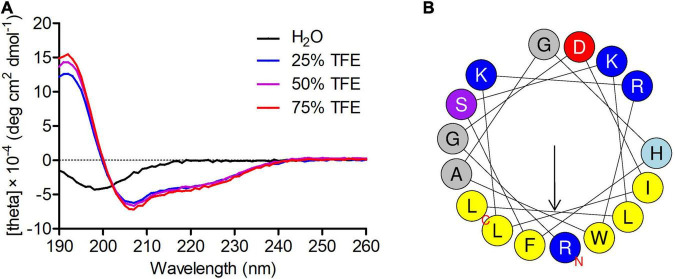
Secondary structure analysis of Phibilin. **(A)** CD spectra of Phibilin (100 μg/mL) in water alone or with 25, 50, or 70% aqueous TFE. **(B)** Helical wheel diagram of Phibilin determined by the Heliquest method.

Circular dichroism spectral analysis showed that in an appropriate membrane environment, Phibilin has an amphipathic helical structure. As shown in Figure 1A, Phibilin exhibited a large negative peak only at about 198 nm in water, indicating a random coil structure, and exhibited a large positive peak at about 195 nm and large negative bands at about 208 and 220 nm in TFE, indicating the predominance of α-helices (Ranjbar and Gill, 2009). The Heliquest program revealed that the α-helical secondary structure of Phibilin is divided into two parts: the hydrophobic face and the hydrophilic face (Figure 1B), consistent with the structural characteristics of amphipathic α-helical peptides (Brogden, 2005). Thus, Phibilin is a typical amphipathic α-helical peptide.

### Antifungal Activity of Phibilin Against *Candida albicans*

Susceptibility assays (Table 2) revealed that Phibilin inhibited the growth of several *C. albicans* strains, including clinical resistant strains. The MIC and MFC values of Phibilin against *C. albicans* were 50 and 100 μg/ml, respectively.

**TABLE 2:** *In vitro* antifungal activities of Phibilin against *C. albicans.*

Strains	MIC		MFC	
		
	μg/mL	μM	μg/mL	μM
*Candida albicans* AY 93025	100	52.7	100	52.7
*Candida albicans* ATCC 10231	100	52.7	100	52.7
*Candida albicans* CMCC 98001	100	52.7	100	52.7
*Candida albicans* clinical resistant strain CR-1	50	26.4	100	52.7
*Candida albicans* clinical resistant strain CR-2	100	52.7	100	52.7
*Candida albicans* clinical resistant strain CR-3	100	52.7	100	52.7
*Candida albicans* clinical resistant strain CR-4	50	26.4	100	52.7
*Candida albicans* clinical resistant strain CR-5	100	52.7	100	52.7

### Phibilin Rapidly Kills *Candida albicans* Cells

**FIGURE 2:**
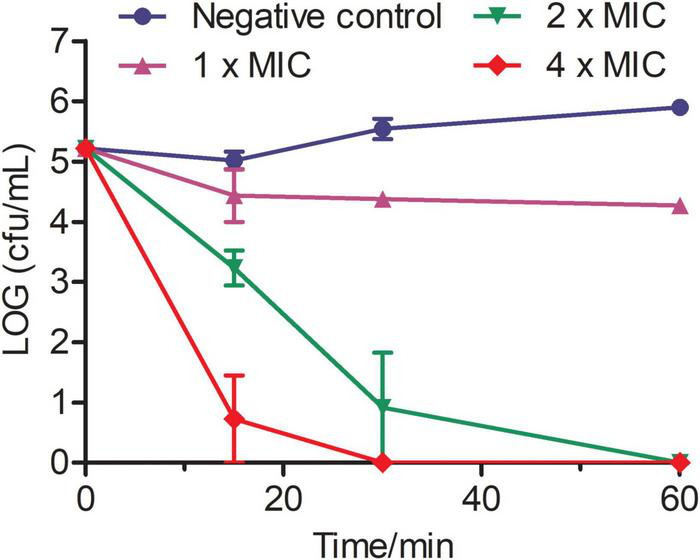
Time-killing kinetics of Phibilin against *C. albicans*. The assay was performed by determining the counts of the surviving cells. The term 0 min represents cells before treatment. Negative control: 0.9% saline.

Time-killing assays performed to investigate the action mode of Phibilin revealed that the killing effect of Phibilin was in a concentration-dependent manner (Figure 2). The *C. albicans* cells were reduced by about 2 log units within just 15 min of treatment with 2 × MIC of Phibilin and by more than 4 log units within 15 min at 4 × MIC of Phibilin treatment. These findings indicated that Phibilin killed *C. albicans* cells rapidly.

### Phibilin Damages *Candida albicans* Plasma Membrane

**FIGURE 3:**
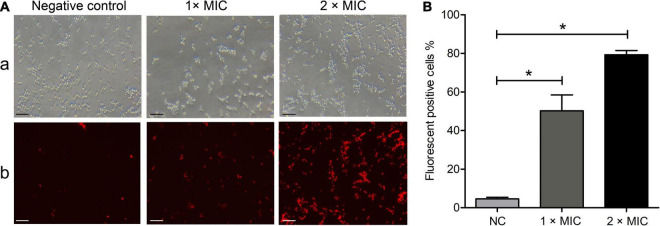
PI absorption assay. **(A)** Fluorescence microscope. a: Bright field; b: Fluorescence field. **(B)** Percentage of the fluorescence-positive cells. NC: Negative control, 0.9% saline. *P < 0.05. Bar: 20 μm.

Membrane-permeabilizing analyses using PI were performed to investigate the influence of Phibilin on the plasma membrane integrity of *C. albicans* cells. As shown in Figure 3, the number of fluorescence-positive cells increased with Phibilin concentration (50.2% at 1 × MIC and 79.3% at 2 × MIC). The percentage of the fluorescence-positive cells significantly increased (*P* < 0.05) with Phibilin treatment when compared to the negative control (4.6%) (Figure 3B). These findings demonstrated that Phibilin damages the plasma membrane of *C. albicans* cells.

### Scanning Electron Microscopy

**FIGURE 4:**
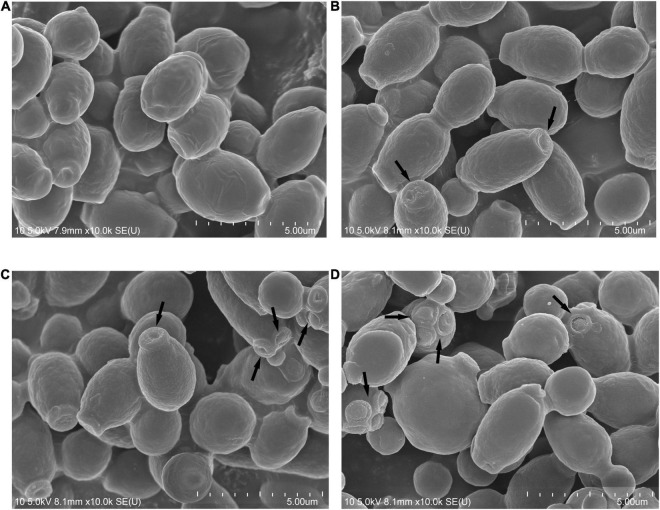
Scanning electron microscopy. **(A)** Negative control, 0.9% saline; **(B)** Treated with Phibilin at 1 × MIC for 30 min; **(C)** Treated with Phibilin at 2 × MIC for 30 min; **(D)** Treated with Phibilin at 4 × MIC for 30 min. Arrow: budding sites of *C. albicans* cells.

Scanning electron microscopy was used to observe the morphological changes in Phibilin-treated *C. albicans* cells. As shown in Figure 4, *C. albicans* cells in both the control group (Figure 4A) and the group treated with Phibilin (Figures 4B–D) contained smooth-walled spherical bodies. However, Phibilin damaged the budding sites of *C. albicans* cells (Figures 4B–D). Thus, Phibilin did not damage the general morphology of *C. albicans* cells but might disrupt the budding of *C. albicans* cells.

### Phibilin Interacts With DNA

**FIGURE 5:**
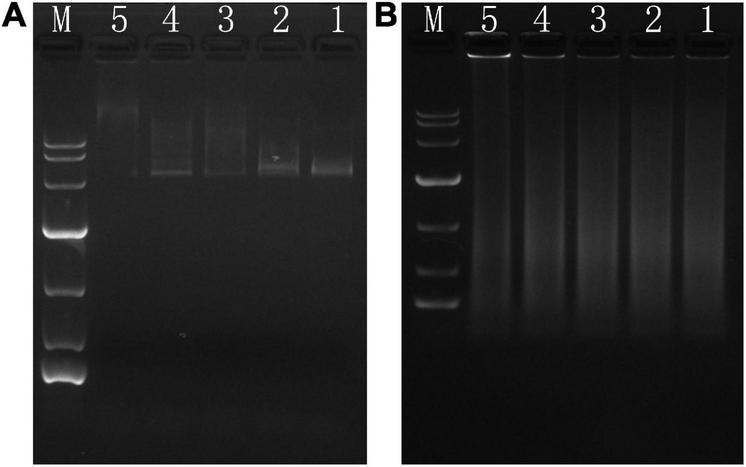
DNA-binding assay. **(A)** pet28a. **(B)** Salmon sperm. Ratio of peptide/DNA: line 5 (40:1), line 4 (20:1), line 3 (10:1), line 2 (5:1), line 1 (0:1).

Gel retardation assays were used to study the DNA-interacting ability of Phibilin. As shown in Figure 5, significant retardation was observed in the double-stranded closed-loop DNA (Figure 5A) and single-stranded linear DNA (Figure 5B). The retardation of the DNAs was dependent on the dose of Phibilin, clearly demonstrating a DNA/Phibilin interaction did not depend on the DNA structure.

### Phibilin Increases ROS Levels in *Candida albicans*

**FIGURE 6:**
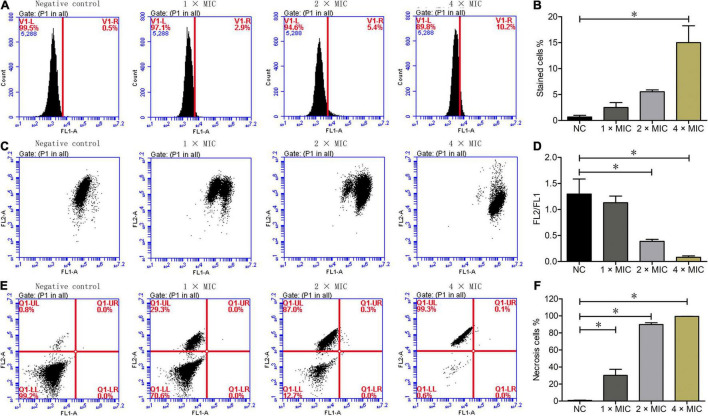
Flow cytometry assays. **(A)** ROS measurement. **(B)** Percentage of DCFH-DA stained cells. **(C)** Mitochondrial membrane potential assays. FL2: Aggregates JC-1; FL1: JC-1 monomer. **(D)** Ratio of FL2/FL1. **(E)** Annexin V and PI staining. FL1-A: Annexin V-FITC; FL2-A: PI. Q1-UL: Necrotic cells (Annexin V-/PI +); Q1-UR: Late apoptotic cells (Annexin V + /PI +); Q1-UR: Early apoptotic cells (Annexin V + /PI-); Q1-LL: Normal live cells (Annexin V-/PI-). **(F)** Percentage of necrotic cells. NC: Negative control, 0.9% saline. *P < 0.05.

To determine whether the killing effect of Phibilin on *C. albicans* cells was ROS-related, the ROS levels in *C. albicans* cells treated with or without Phibilin were analyzed by flow cytometry. As shown in Figures 6A,B, Phibilin treatment increased the ROS levels in *C. albicans* cells in a concentration-dependent manner, indicating the ROS accumulation in *C. albicans* cells induced by Phibilin.

### Phibilin Disrupts the Mitochondrial Membrane Potential of *Candida albicans*

To determine the influence of Phibilin on the mitochondrial membrane potential of *C. albicans* cells, a JC-1 staining assay was performed. As shown in Figures 6C,D, Phibilin treatment decreased the FL2/FL1 ratio in a dose-dependent manner, suggesting a decrease in the mitochondrial membrane potential after Phibilin treatment. Thus, Phibilin-induced oxidative stress caused the depolarization of the mitochondrial membrane of *C. albicans*.

### Phibilin Induces Necrosis of *Candida albicans* Cells

To further assess if the killing effect of Phibilin on *C. albicans* cells induces cell apoptosis and/or necrosis, *C. albicans* cells were labeled with Annexin V and PI after treatment with or without Phibilin, followed by flow cytometry assay. As depicted in Figures 6E,F, necrosis occurred in cells in the V–/PI + (Q1-UL) quadrant, late apoptosis occurred in the Annexin V + /PI + (Q1-UR) quadrant, early apoptosis occurred in the Annexin V + /PI– (Q1-LR) quadrant, and there was no change in the cells in the Annexin V–/PI– (Q1-LL) quadrant. Phibilin treatment increased the number of cells in the Annexin V–/PI + (Q1-UL) group, whereas those in the Annexin V + /PI + (Q1-UR) and Annexin V + /PI– (Q1-UR) quadrants remained almost unchanged, indicating that Phibilin could induce *C. albicans* necrosis. These findings indicated that Phibilin exerted its antifungal activity by directly killing *C. albicans* cells.

### Phibilin Affects *Candida albicans* Biofilms

**FIGURE 7:**
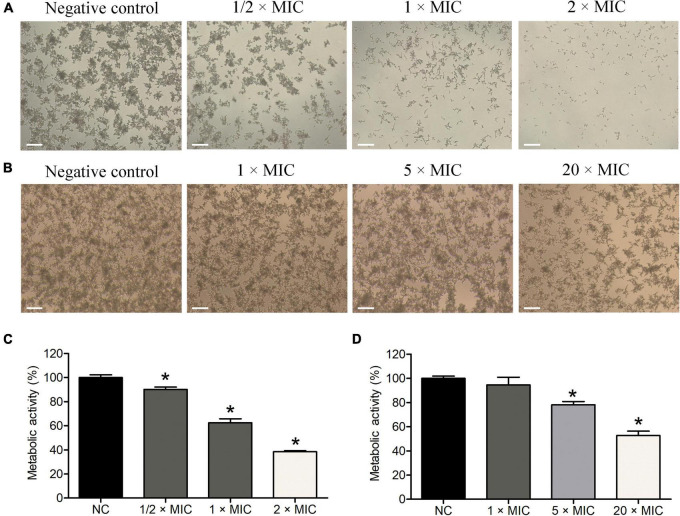
Effects of Phibilin on biofilms of *C. albicans*. Inhibitory effects of Phibilin on biofilm formation investigated by microscope **(A)** and XTT assay **(C)**. Reduction effects of Phibilin on mature biofilms investigated by microscope **(B)** and XTT assay **(D)**. NC: negative control, 0.9% saline. *P < 0.05. Bar: 20 μm.

Biofilms are crucial for *C. albicans* infections. As shown in Figures 7A,C, Phibilin significantly inhibited the formation of *C. albicans* biofilm in a dose-dependent manner. The Phibilin concentrations of 1/2 × MIC, 1 × MIC, and 2 × MIC decreased the formation of *C. albicans* biofilm by 9.85% (*P* < 0.05), 37.53% (*P* < 0.05), and 61.50% (*P* < 0.05), respectively. Also, Phibilin reduced the completely formed biofilms of *C. albicans* in a dose-dependent manner (Figure 7B). In particular, 1 × MIC, 5 × MIC, and 20 × MIC of Phibilin reduced the biofilm formation in *C. albicans* by 5.45% (*P* > 0.05), 21.94% (*P* < 0.05), and 47.35% (*P* < 0.05), respectively (Figure 7D). These findings indicated that Phibilin not only inhibited the biofilm formation but also reduced the mature biofilms of *C. albicans*.

### Effects of Phibilin on *Candida albicans* Hyphal Formation

**FIGURE 8:**
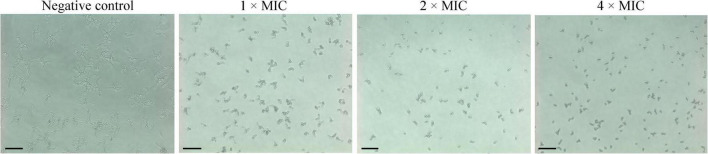
Effect of Phibilin on the hyphal growth of *C. albicans*. Negative control: 0.9% saline. Bar: 20 μm.

Hyphae are important virulent factors that participate in the pathogenesis of *C. albicans*. As displayed in Figure 8, *C. albicans* formed dense and long hyphae in the negative control group. However, Phibilin significantly reduced the normal growth of *C. albicans* hyphae in a dose-dependent manner. These results indicated that Phibilin inhibits yeast-to-hyphal transformation in *C. albicans*.

### Drug Interaction Interpretation

The interaction between Phibilin and clotrimazole, amphotericin B, nystatin, or anidulafungin was investigated using the checkerboard assay. As presented in Table 3, a combination of Phibilin/clotrimazole displayed synergistic activity against *C. albicans*. The FICI value for this combination was 0.375, whereas the rest of the combinations with FICI values ranging from 0.75 to 1.25 showed no synergistic effect. Thus, the combination of Phibilin/clotrimazole could be used as an effective treatment against skin infections caused by *C. albicans*.

**TABLE 3:** Synergistic effects of Phibilin in combination with antifungal agents against *C. albicans.*

Parameters	Clotrimazole	Amphotericin B	Nystatin	Anidulafungin
MIC	10 μg/mL	5 μg/mL	5 μg/mL	0.01 μg/mL
FICI	0.375	1.25	0.75	0.75

### Phibilin Has Low Hemolytic Activity and Cytotoxicity

**FIGURE 9:**
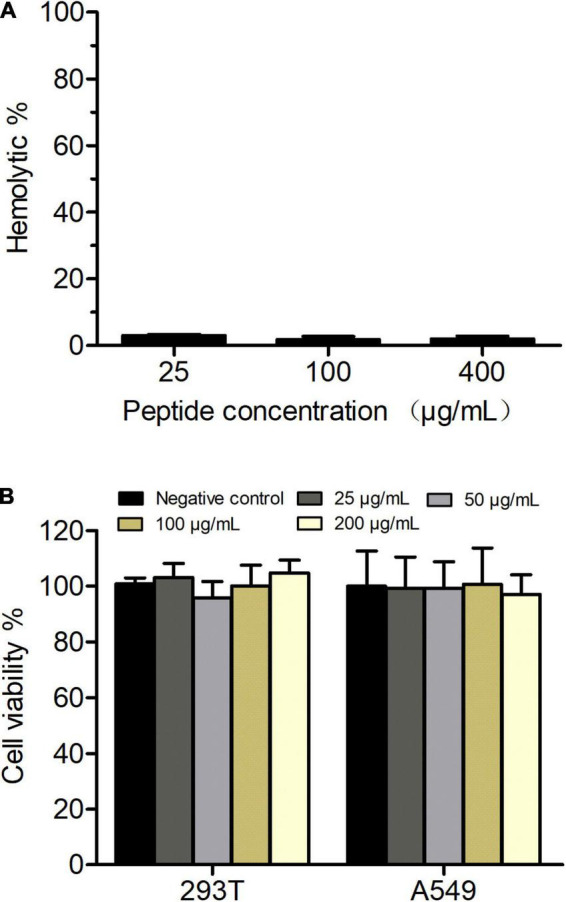
*In vitro* hemolytic activity and cytotoxity of Phibilin. **(A)** The hemolytic activities of Phibilin were evaluated on fresh mouse red blood cells. **(B)** Cytotoxicity effects of Phibilin against HEK293T and A549 cell lines.

As shown in Figure 9A, a Phibilin concentration of 400 μg/ml only slightly hemolyzed the red blood cells (2%). The MTS assay further revealed that a 200 μg/ml concentration of Phibilin was non-cytotoxic to HEK293T and A549 cells (Figure 9B). These results showed that Phibilin exhibited low toxicity.

### Phibilin Inhibited *Candida albicans In vivo*

**FIGURE 10:**
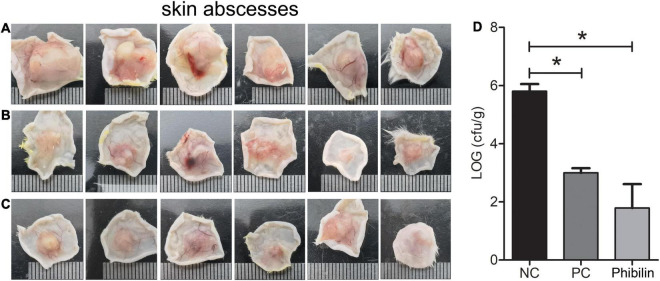
*In vivo* activity of Phibilin against *C. albicans*. The *in vivo* activity of Phibilin was tested in a mouse cutaneous infection model induced by *C. albicans* AY93025. **(A)** Skin abscesses from the negative control group; **(B)** Skin abscesses from the positive control group; **(C)** Skin abscesses from Phibilin treatment group; **(D)** The number of CFU per gram of the tissue. NC: Negative control group, 0.9% saline. PC: positive control group, clotrimazole (500 μg/ml). *P < 0.05.

A mouse model of cutaneous infection with *C. albicans* was used to investigate the activity of Phibilin *in vivo*. As shown in Figure 10, skin abscesses in the Phibilin treatment group (Figure 10C) and positive control group (Figure 10B) were significantly lower when compared to the negative control group (Figure 10A). Furthermore, the number of *C. albicans* cells decreased significantly (*P* < 0.05) after treatment with Phibilin, and the decrease was comparable to the group treated with commercial drugs (*P* > 0.05). These results suggest that Phibilin prevents the formation of skin abscesses and kills *C. albicans* cells *in vivo*.

## Discussion

*Candida albicans* is a commensal fungus of the human microbiome but can cause severe mucosal infections and fatal invasive infections in immunocompromised individuals (Alby et al., 2009; Ganguly and Mitchell, 2011). The rapid increase and emergence of drug-resistant *C. albicans* infections underscore the need to develop effective alternative agents against this fungus. AMPs are found to be potential candidates for the development of new antifungal drugs (Buda De Cesare et al., 2020). In this study, Phibilin, a new AMP identified from *P. bilineatus*, showed antimicrobial activity against *C. albicans*, including the clinical resistant strains (Table 2), with low hemolytic activity and cytotoxicity (Figure 9).

Phibilin is a typical amphipathic α-helical peptide (Figure 1). AMPs with amphipathic α-helical structures are membrane-lytic peptides that kill bacterial and fungal cells (Hancock, 2001; Yang et al., 2001; Brogden, 2005; Hartmann et al., 2010; Huang et al., 2010). Further analyses demonstrated that Phibilin exhibits this property against *C. albicans* cells (Figure 2). PI absorption assay showed that Phibilin exerts its killing effect by disrupting the integrity of the plasma membrane of *C. albicans* (Figure 3), implying that Phibilin is a fungicidal membrane-acting AMP. Besides disrupting the cell membranes, AMPs could also disrupt the morphology of microbial cells, such as CKR12-PLGA (Mori et al., 2021). However, the scanning electron microscopy showed that Phibilin had no effect on the general morphology of *C. albicans* cells (Figures 4B–D). Due to the cationic property of most of the membrane-acting AMPs, they can interact with specific and vital anionic components in the microorganisms, especially nucleic acids, upon penetrating the membrane (Phadke et al., 2003; Soman et al., 2009; Sousa et al., 2016; Wang et al., 2017), such as APP (Li et al., 2016b) and P7 (Li et al., 2016a). In addition to being a membrane-acting AMP, Phibilin is also a cationic antimicrobial peptide. To determine whether Phibilin could interact with nucleic acids, a DNA-binding assay was performed. We found that Phibilin interacts directly with DNA, regardless of the DNA structure (Figure 5), indicating the occurrence of only electrostatic interactions between Phibilin and DNA.

Even though the mitochondria generate ROS, high levels of ROS depolarize the mitochondrial cell membrane (Terman et al., 2006; Pereira et al., 2008). The accumulation of ROS and depolarization of mitochondrial membrane play an essential role in the apoptosis of cells (Ott et al., 2007; Farrugia and Balzan, 2012). Usually, fungicidal drugs induce apoptosis by increasing the production of ROS (Hwang et al., 2012; Belenky et al., 2013), such as the AMP PMAP-23 (Kim and Lee, 2019), pleurocidin (Cho and Lee, 2011), and hepcidin-25 (Chen and Lan, 2020). As a fungicidal peptide, Phibilin might also kill *C. albicans* cells in a similar manner. Further analyses revealed that Phibilin induces the accumulation of ROS in *C. albicans* cells in a dose-dependent manner (Figures 6A,B), which also decreased the mitochondrial membrane potential in a dose-dependent manner (Figures 6C,D). The effect of Phibilin on ROS is shared by other AMPs, such as PMAP-23 (Kim and Lee, 2019), pleurocidin (Cho and Lee, 2011), and hepcidin-25 (Chen and Lan, 2020). Thus, Phibilin killed *C. albicans* in a ROS-dependent manner. In addition, Phibilin caused necrosis and not apoptosis of *C. albicans* (Figures 6E,F), which is different from the aforementioned AMPs. However, the necrotic activity was comparable to that of other AMPs, such as VS2 and VS3 (Maurya et al., 2011), MK58911 (Singulani et al., 2019), and the antifungal agent 4-AN (Martyna et al., 2020). Taken together, Phibilin killed *C. albicans* cells by disrupting the integrity of the plasma membrane and triggering necrosis of the fungi *via* a ROS-related pathway.

*Candida albicans* can form biofilms that are difficult to be damaged. Given its critical role in *C. albicans* infections, the biofilm enhances resistance of *C. albicans* to antifungal agents and host defenses, undermining the effective treatment of this infection (Khan et al., 2017; Wall et al., 2019). Further analyses showed that Phibilin not only significantly inhibited the biofilm formation of *C. albicans* but also reduced the mature biofilms in a dose-dependent manner (Figure 7). Other AMPs, such as dermaseptin-S1 (Belmadani et al., 2018) and polybia-MPI (Wang et al., 2014), display comparable properties. As a dimorphic fungus, *C. albicans* displays two morphological forms of yeast and hyphae (Pereira et al., 2021). The yeast-to-hyphal transition is crucial for biofilm formation and enhances the pathogenesis and drug resistance of the fungus (Pozzatti et al., 2010; Lu et al., 2014; Nobile and Johnson, 2015; Noble et al., 2017). We found that Phibilin significantly inhibits hyphal formation (Figure 8). The yeast-to-hyphal transformation of *C. albicans* is also known as a budded-to-hyphal-form transition (Atriwal et al., 2021). We hypothesized that Phibilin affects the budding of *C. albicans*. Scanning electron microscopy confirmed that Phibilin destroyed the budding sites of *C. albicans* cells (Figures 4B–D), a property displayed by other AMPs, such as GMAP (Rauch et al., 2007) and LL13–37 (Wong et al., 2011). Thus, Phibilin inhibited the yeast-to-hyphal transformation of *C. albicans* cells by destroying the budding sites, which subsequently affects the formation of biofilms.

The combination therapies reduce the dosage and duration of drug treatment and the associated side effects, show maximum antimicrobial effects, and in particular prevent or alleviate the development of resistance (Chen et al., 2021). Thus, it is interesting to know whether there is a synergistic relationship between Phibilin and antifungal agents. We found that Phibilin and clotrimazole displayed a synergic effect against *C. albicans* (Table 3). However, there were no antagonistic or synergistic effects between Phibilin and amphotericin B, nystatin, and anidulafungin (Table 3). Thus, a combination of Phibilin and clotrimazole can effectively treat skin infections caused by *C. albicans*.

The loss of activity *in vivo* and high systemic toxicity limit the application of AMPs for treating microbial infections, and most of them are studied for unsystematic applications (Fjell et al., 2011; Pfalzgraff et al., 2018; Thapa et al., 2020). Although Phibilin displayed a low hemolytic activity and cytotoxicity (Figure 9), we investigated the effect of Phibilin *in vivo* using a mouse cutaneous infection model induced by *C. albicans*. We found that Phibilin prevented the formation of skin abscesses and decreased the CFU of *C. albicans* in the tissue (Figure 10). Moreover, no significant difference was observed in the effect of Phibilin when compared to the treatment group acting as positive drug control. In general, our findings indicate that Phibilin is a potential antifungal agent that is effective against skin infections caused by *C. albicans*.

## Conclusion

Phibilin is a new AMP that is effective against *C. albicans* infections, including the clinical resistant strains. It also displayed synergistic effects with clotrimazole. Phibilin killed *C. albicans* cells mainly by damaging the cell membrane, interacting with DNA, and inducing necrosis of *C. albicans* cells *via* ROS-related pathway. Moreover, Phibilin inhibited the biofilm formation of *C. albicans* by preventing hyphal development in a dose-dependent manner. Mouse skin infection models revealed that Phibilin still showed excellent antifungal activity against *C. albicans in vivo*. Overall, Phibilin is a potential anti-infective agent against skin infections caused by *C. albicans*.

## Data Availability Statement

The original contributions presented in the study are included in the article/supplementary material, further inquiries can be directed to the corresponding author/s.

## Ethics Statement

The animal study was reviewed and approved by all animal experiments were conducted following the Animal Care Ethics guidelines with protocols approved by the Animal Care and Use Committee of Henan University of Technology and Science.

## Author Contributions

ZL, SL, WZ, and BD designed the study and prepared the manuscript. ZL and SL analyzed the data. ZL and WZ revised the manuscript. ZL and XJ provided financial support for the conduct of the research. All authors performed all the experiments.

## Conflict of Interest

The authors declare that the research was conducted in the absence of any commercial or financial relationships that could be construed as a potential conflict of interest.

## Publisher’s Note

All claims expressed in this article are solely those of the authors and do not necessarily represent those of their affiliated organizations, or those of the publisher, the editors and the reviewers. Any product that may be evaluated in this article, or claim that may be made by its manufacturer, is not guaranteed or endorsed by the publisher.

## Footnotes

1
http://www.camp.bicnirrh.res.in/

2
http://heliquest.ipmc.cnrs.fr/cgi-bin/ComputParams.py
